# Lessons Learnt during the Implementation of WISN for Comprehensive Primary Health Care in India, South Africa and Peru

**DOI:** 10.3390/ijerph182312541

**Published:** 2021-11-28

**Authors:** Sikhumbuzo A. Mabunda, Mona Gupta, Wezile W. Chitha, Ntombifikile G. Mtshali, Claudia Ugarte, Ciro Echegaray, María Cuzco, Javier Loayza, Felipe Peralta, Seimer Escobedo, Veronica Bustos, Onke R. Mnyaka, Buyiswa Swaartbooi, Natasha Williams, Rohina Joshi

**Affiliations:** 1 The George Institute for Global Health, University of New South Wales, Sydney 2042, Australia; rjoshi@georgeinstitute.org; 2 School of Population Health, University of New South Wales, Sydney 2033, Australia; 3 National Health Systems Resource Centre, Ministry of Health and Family Welfare, New Delhi 110067, India; monagupta@outlook.com; 4 Health Systems Enablement & Innovation Unit, University of the Witwatersrand, Johannesburg 2000, South Africa; wezile.chitha@wits.ac.za (W.W.C.); onkemnyaka@gmail.com (O.R.M.); bswaartbooi@witshealth.co.za (B.S.); nwilliams@witshealth.co.za (N.W.); 5 School of Nursing and Public Health, University of KwaZulu-Natal, Durban 4041, South Africa; mtshalin3@ukzn.ac.za; 6 General Directorate of Health Personnel, Ministry of Health, Lima 15072, Peru; claudia.ugarte@upch.pe (C.U.); cechegarayp@minsa.gob.pe (C.E.); mcuzco@minsa.gob.pe (M.C.); cloayza@minsa.gob.pe (J.L.); fperaltaq@unmsm.edu.pe (F.P.); 7 Department of Medical Clinics, Universidad Peruana Cayetano Heredia (UPCH), Lima 15102, Peru; 8 Department of Preventive Medicine and Public Health, Universidad Nacional Mayor de San Marcos (UNMSM), Lima 15081, Peru; 9 Health Systems, Peruvian Society of Health Administration, Lima 15046, Peru; sescobedope@yahoo.com; 10 Human Resources for Health, Independent Consultant, Lima 15074, Peru; verobustosf@hotmail.cl; 11 The George Institute for Global Health, New Delhi 110025, India

**Keywords:** WISN, decision making, health workforce, planning, health systems, health policy

## Abstract

Introduction: The World Health Organization introduced the workload indicators of staffing needs (WISN) in 1998 to improve country-level health workforce planning. This study presents the primary care health workforce planning experiences of India, South Africa and Peru. Methods: A case study approach was used to explore the lessons learnt in the implementation of WISN in India and South Africa. It also describes the methods developed and implemented to estimate health workforce in Peru. We identify the barriers and facilitators faced by countries during the implementation phase through the triangulation of literature, government reports and accounts of involved health planners in the three countries. Results: India implemented WISN in a referral pathway of three district health facilities, including a primary health centre, community health centre and district hospital. Implementation was impeded by limited technical support, poor stakeholder consultation and information systems challenges. South Africa implemented WISN for health workforce planning in primary care and found the skills mix and staff determinations to be unaffordable. The Peruvian Ministry of Health considered using WISN but decided to develop a context-specific tool to estimate the health workforce needed using its available resources such as the National Register of Health Personnel. The main challenge in using WISN was the insufficient information on its inputs. Conclusion: While India and South Africa had unique experiences with the integration of WISN in their health system, none of the countries has yet benefited from the implementation of WISN due to financial, infrastructure and technical challenges. Since the methodology developed by the Peruvian Ministry of Health is context-specific, its implementation has been promising for health workforce planning. The learnings from these countries’ experiences will prove useful in bringing future changes for the health workforce.

## 1. Introduction

In order to plan and deliver health services optimally, health systems need to estimate the number and type of human resources needed in the short, medium and long term [1,2]. The health workforce planning process starts off with the identification of key socio-demographic and/or epidemiologic and/or economic and/or legislative and/or environmental variables to determine future health workforce requirements [3,4]. The context, capacity and availability of data will ultimately determine the planning approach used [4]. For instance, in contexts where there is a lack of capacity and/or data, the simplest method used is the workforce-to-population ratio method [4]. This is an unsophisticated strategy that assumes homogeneity at the level of the numerator (e.g., nurses) and the denominator (population needs are similar and constant) [4]. This contrasts more in-depth approaches, such as the health needs method, the service demands method and the service target method [4]. The health needs method projects health workforce needs based on current and anticipated changes in population needs for health services and thus relies on demographic, sociocultural and epidemiological data [4]. The service demands method is based on available information on health service utilisation rates for different population groups and applies these rates to the future population profile to determine the health workforce competencies required to service their needs [4]. Lastly, the service target method presumes service utilisation and specifies targets for the production of various types of health services and the defined packages of care of that health facility based on a set of assumptions [4]. The strategy determines how the services must evolve in number, size and staffing in accordance with productivity norms [4].

To complement these approaches, several tools and innovations have been used to support health workforce planning and projections. These include the workload indicators of staffing needs (WISN), trend analysis, regression analysis, meta-analysis, econometric analysis and disease-specific models, e.g., HIV [3,4]. There are other context-specific tools available, such as England’s robust workforce planning framework, New Zealand’s workforce intelligence and planning framework and Australia’s health workforce planning tool [3]. It is important to acknowledge that most of the inaccuracies in health workforce projections often arise due to planning exercises that simply project forward the status quo and fail to account for future demographic, epidemiological, social, environmental and economic changes [3,5].

The World Health Organization (WHO) developed WISN, a method to calculate human resources needs at the primary, secondary and tertiary level health facilities, considering the health delivery models and population demands [6]. It provides two indicators to assess staffing needs (a) the gap between current and required number of staff and (b) WISN ratio, a measure of workload pressure on health workers [7]. WISN was developed in 1998 and has been field tested in several countries [1]. However, some countries have not been able to implement it due to challenges in the availability of workforce and workload data which are essential inputs for WISN [7]. Countries have also faced challenges in using the WISN software [7], while some countries who used WISN to calculate human resources for health have not been able to use the recommendations as they could not afford the number of staff calculated by WISN [7]. Finally, countries such as Peru developed an innovative method to calculate human resources for health.

In this paper, we aim to describe the lessons learnt during the implementation of WISN in India and South Africa and new methods developed by Peru. Each of these countries represents diverse populations facing the dual burden of communicable and non-communicable diseases, recent health system reforms and varying health workforce shortages.

## 2. Methods

### 2.1. Design and Study Setting

We used a case study approach [4,8] to explore the lived experiences of government officials and academics regarding the lessons learnt in the implementation of WISN for primary care health workforce planning in Peru, India and South Africa. This article reflects three case scenarios from the three aforementioned countries. The study included discussions with policymakers and academics who were involved in the implementation of WISN and/or health workforce planning. A case study approach is useful to obtain and present a multi-faceted understanding of a complex issue [8]. Each case study described in this paper includes a brief overview about the socio-economic context of the country and health system reforms gleaned through professional networks comprising of government officials and researchers with interest and/or involvement in health workforce planning. Second, we undertook a literature review of the implementation of WISN in the three countries. Third, we captured the lived experiences from the lens of government officials and academics involved in WISN implementation in the three countries. The case studies presented give an overview of how WISN was implemented and the key lessons learnt during the process. We identified the key barriers and facilitators faced by countries during the implementation phase through the triangulation of literature, government reports and accounts of involved health planners in the three countries.

These countries in three different continents and WHO regions; namely, WHO South-East Asia Region (WHO-SEARO), WHO Africa and Pan American Health Organization (PAHO), present diverse views and experiences with WISN implementation and uptake. While all three countries are middle-income and are undergoing health reforms towards universal health coverage, they also have similar disease burdens and human resource challenges. They were therefore conveniently selected as a representation of their continent and region.

### 2.2. Patient and Public Involvement

No-one other than the authors who represent the three countries was interviewed. Patients and members of the public were, therefore, not involved in the design and execution of this study.

### 2.3. Ethics Approval

Ethical approval is not required for this study as it is a report on the implementation experiences of the authors.

## 3. Results

Table 1 gives an overview of the country context of the three countries.

### 3.1. India

#### 3.1.1. Country Context

India has a population of 1.38 billion with variations in geographical terrain, demography, culture, income and many others, including health-seeking behaviour and health indicators. India’s federal structure comprises of 28 states and 8 union territories, further subdivided into 719 districts for administrative purposes [13,14].

#### 3.1.2. Health System Context

Health is a state subject under the Indian constitution, though the central government funds a major part of some national programs and drives reforms through these programs [15,16,17,18,19,20]. India has a tiered system of healthcare, including public and private healthcare [16]. The public sector includes primary, secondary and tertiary health centres [16]. In the past 30 years, India has seen rapid economic development which has brought changes in lifestyle and healthcare [11,13,14]. The average life expectancy at birth has increased from 58.7 years in 1990 to 69.4 years in 2019 [11,13,14]. The concurrent epidemiological transition has seen a slight decline in mortality due to communicable, maternal, neonatal and nutritional diseases and an increase in disease burden due to non-communicable diseases and injuries [13,14,16,19]. In April 2018, India embarked on the journey of comprehensive primary health care which aspires to transform 150,000 sub-centres and primary health centres into health and wellness centres by 2022 [13,14,15,16,17,19,20].

The availability of the health workforce has significantly improved in the last decade, with the country producing 80,000 generalist doctors, 48,000 medical specialists and 320,000 nurses and auxiliary nurses every year [18,19]. The staffing in the public health facilities is expected to be as per the Indian public health standards, which is based on expert consultations [18,19]. Though the workload should ideally be considered while planning, it is not widely practiced.

#### 3.1.3. Use of WISN

Planning: With the objective of bringing evidence into the health workforce planning process, a pilot study including three facilities which form part of the public health referral chain (primary health centre, community health centre and district hospital) was initiated in 2018 in one of the districts in a north Indian state which is representative of an average high focus group state (states prioritised by the government due to weaker health systems and indicators). While the WISN methodology has evolved overtime and the guidelines recommend steps to ensure smooth assessment, the concepts and their importance are not apparent when implementing WISN for the first time.

Implementation: The pilot study, which was to be completed in six months, could not be concluded successfully. The key challenges faced in piloting are grouped into the following:

(1) Inadequate stakeholder consultation: An orientation meeting with all the staff and policymakers involved to explain the need for and importance of WISN was not carried out. Instead, the process relied solely on one-on-one discussions. With the central team conducting the study in district and sub-district facilities, the process was seen as a ‘top-down’ approach. Past, unsuccessful attempts of resolving issues including staffing needs (without WISN) made some staff sceptical about such exercises. The district health administrators and the medical specialists were reluctant to be part of the committees which were to be set up for deciding standard working time and providing technical oversight, citing work pressures. Though the WISN methodology advocates for three committees, these were seen as far too many for the district. Furthermore, staff were not convinced that it was worth their time and effort.

In the past, WISN was used in a stand-alone hospital with focus on a single cadre and/or for a single programme (e.g., maternal and child health services) by non-governmental stakeholders [15,17,20]. However, we could not find any updated documentation or evidence of its incorporation in policy documents. Discussion with officials revealed that the main reason was inadequate involvement of all the stakeholders who would later implement the findings.

(2) Issues in defining workload components and standard time: Without prior knowledge or experience in conducting WISN, classifying workload components and devising standard time proved difficult for the team. The national Health Management Information System (HMIS) captures health service activities but does not and is not expected to capture other supporting tasks and additional activities. Capturing data on such tasks can only be done through observation, making WISN time and resource intensive. As it involved observing each of the main cadres for at least three days, it felt like an intrusion and was resented by some staff.

The team realised that the presence of support staff vastly reduced the time taken. For instance, during a surgical procedure, someone checks the vital signs, the anaesthetist performs the pre-operation check, the nurse prepares the patient for theatre, while another prepares the theatre table, etc. If WISN calculations for a surgeon are done without incorporation of the team members, the surgeon’s workload will be inflated. Hence there is a need to capture all cadres in WISN or, alternatively, to include all associated in the expert group. The first option would make the WISN exercise too big, whereas the second would involve a higher degree of co-ordination.

(3) Readiness and experience of the piloting team: Availability of the WISN user manual, software manual and WISN software led the team to believe that the study could be done without any external technical support. However, it proved difficult and a time-consuming exercise in the field, e.g., discussions with the staff on additional activities and arriving at individual allowance standard. This led to an awareness that the piloting team would need some handholding from those who had carried out WISN earlier and could resolve the issues based on their past experience with greater efficiency.

Lessons learnt: Ideally, the entire team, including facility teams, should be involved in the planning phase and convinced about the usefulness of WISN. This may take time and may involve more than one round of deliberations. Without adequate stakeholder consultation and creating an environment of trust which comes from the assurance of the leadership at state level, followed by concrete steps towards change, people at facility level may not be convinced that WISN will change or improve anything. Staff at the health facility should have been trained in WISN methods and conducted it themselves with the support of district, state and other technical bodies. India is now planning to pilot WISN in three districts of three states where learnings from the earlier attempt would prove useful in bringing changes in staffing. This time, the key change is ensuring stakeholder consultation where policymakers from central and state levels, as well as facility-level healthcare staff, are involved from the beginning and make the process a ‘bottom-up’ approach with the technical support from experts (e.g., the WISN community of practice) who have implemented WISN successfully.

### 3.2. South Africa

#### 3.2.1. Country context

South Africa is an upper-middle-income country with 59.6 million citizens [10,11]. While approximately 64.7% of the population inhabit the provinces that are largely rural in nature, 43.6% of citizens reside in rural areas [10,21]. South Africa’s health system is structurally formed into nine provincial health administrations and fifty-two health districts [22]. The National Department of Health is responsible for policy; each provincial department of health (PDoH) is responsible for policy implementation; and health districts initiate the delivery of healthcare through a comprehensive primary health care approach [23,24].

#### 3.2.2. Health System Context

The South African constitution guarantees all citizens access to health services [24,25]. However, the health system is fragmented into the public sector and the private sector, which is only accessible to those who can afford it and is mostly in urban areas [23,26]. The District Health Information System (DHIS) is the standard information system used in the public sector [27,28]. Even though the health system consumes about 8.8% of the country’s gross domestic product, only 4.1% goes towards the public sector which serves 84% of the population [23,25,29].

Significant progress has been made in unifying the health system through the revitalisation of primary health care and progress made towards universal health coverage since 2011 [21,23,25,29,30]. However, the country is struggling to meet sustainable development targets due to the quadruple burden of disease, namely, HIV/AIDS and TB, high maternal and child mortality, non-communicable diseases and injuries [31], that is exacerbated by human resource shortages and maldistribution [30,32]. The country has nearly 503 health workers for every 100,000 population [21]. The distribution of the workforce is skewed, with rural areas served by 19% of the nurses and 12% of the doctors [21]. Even though each PDoH is the direct employer of the health workforce, posts are determined by the national Department of Public Service and Administration. Budgets are disseminated equitably to provinces from the national treasury [23,26]. The primary health care policy, implemented since 1994, is set from the National Department of Health and used to ensure a sustained shift towards comprehensive primary health care which is implemented through the district health system [33].

#### 3.2.3. Use of WISN

Planning: In 2012, South Africa took a policy decision to apply WISN for health workforce planning in primary care [34]. This included determining staff numbers and identifying the skills mix required for efficient service delivery [35]. However, the health workforce normative guidelines and standards which provided the legal framework for WISN implementation were only finalised in October 2015 [2,34,35]. Of importance, is that provinces were allowed the flexibility and capacity to offer additional services in order to address the burden of disease in their area [35]. The variance from the normative guide would be assessed at least annually using the WISN tool [35].

Implementation: The national head of health appointed national and provincial WISN coordinators to facilitate implementation across provinces [34]. Each provincial head of health appointed WISN implementation groups including district champions and donor-funded support partners to facilitate implementation at district and local level [34]. Each facility determined its own health workforce needs, but the ultimate responsibility for implementation rested with the district manager, supported by the WISN teams [35]. However, the WISN tool was not installed in all facilities or district offices but largely centred in provincial offices. Facilities populated a paper-based Excel spreadsheet which was edited at sub-district and district level. This is largely because the South African health system is largely paper-based and has struggled to create a reliable information and communication technology (ICT) infrastructure and skills base that could have enabled deployment of the WISN tool to health facilities and districts. Furthermore, DHIS does not assess individual level data, workload and working time data; as a result, these relied on subjective reporting by health facilities [27,28].

WISN coordinators were offered five days of training by WHO health systems experts on WISN implementation in a train-the-trainer model [34,35]. Trained provincial WISN coordinators trained implementers in their respective provinces [34,35]. Participants reported satisfaction with the tool as it informed their health workforce needs [2]. Some users lacked basic computer literacy, thus limiting their efficiency on using the tool [2]. There was sufficient and continuous technical support provided by WHO experts, WISN coordinators and the donor-funded support partners [34].

Post-implementation: The recommended skills mix and HRH projections calculated using WISN were found to be unaffordable [34]. For instance, in one province the WISN determined posts would require an additional ~5.3 billion Rands (ZAR) above what the province could afford (Table 2). The results, therefore, served to inform the gaps and skills mix needed in primary care to provide evidence for decision making but not the strategies to recruit or retain them.

Lessons learnt: WISN-specific limitations faced included dependence on the accuracy of the annual service statistics used to assess workloads; possible over-reporting of annual service statistics; inability to differentiate 40 h per week (8-h weekday) and 24-h-a-day facilities and activities; inability to differentiate when the same activity is performed by multiple staff categories; and insufficient consideration of the unique circumstances and HRH needs in rural areas [36]. The process seems to have come to a halt with failure to implement WISN in hospitals. While affordability is not within the scope of WISN, countries need to understand the gaps in the health workforce to enable better planning [6].

Lack of consultation during implementation [34] highlighted the importance of broader stakeholder consultation, e.g., the National Treasury and the Department of Public Service, for the successful implementation of the findings. Consequently, South Africa’s 2030 HRH strategy published in mid-2020 does not make use of WISN for long-term HRH planning in primary care but, instead, uses a method that relies on the utilisation of services [30,37]. This model relies on the use of a primary care utilisation rate of 3.2 visits per person per year then calculates the number of primary care staff required to provide those visits to the uninsured population using data from a staffing time survey [30,37].

Recommendations: In addition to identifying the staff need and skills mix, WISN could be used to model the staffing requirements and model their realisation over time within available resources. Consultations with organised labour and engagement with key stakeholders such as the national treasury, department of public service and administration and provincial departments of health are more likely to embrace evidence-based health workforce outputs that are deemed to be necessary to the degree that the country can afford. Improving computer literacy amongst health workers, strengthening facility ICT infrastructure and information management can enable decentralisation of the tool at the facility level.

### 3.3. Peru

#### 3.3.1. Country Context

The Republic of Peru in South America has twenty-six political regions distributed along the coastline and in the mountains and forest, with approximately 78% of its 32.8 million population living in urban regions [11,38,39,40,41].

#### 3.3.2. Health System

The Peruvian health system is fragmented, segmented and serviced by the public and private sector [38,39,40,41,42,43]. Approximately 60% of the health expenditure is drawn from the Ministry of Health (MINSA), 30% from the social health insurance (EsSalud) system, and the remaining (10%) is from the armed forces, national police and private sector [38,39,40,41,42,43]. Universal health insurance was passed in 2009 and now 80% of the Peruvian population has health insurance [38,39,40,41,42,43]. The health system is currently in the process of shaping the integrated health networks within the framework of the comprehensive health care model by life-course (MCI) [44,45]. According to the Observatory of Human Resources for Health, as of October 2020 [46], Peru’s health workforce included more than 330,000 personnel. The majority (76%) of the workforce are employed by the Ministry of Health (MINSA) and regional governments [38,39,40,41,42,43,44,45]. The density of human resources for health in Peru is 38.44 per 10,000 population, corresponding to doctors at 14.9 per 10,000 population with a range of 8.14 to 22.82 [11,38,39,40,41,42,43,44,45].

#### 3.3.3. Use of WISN

Planning: The Peruvian Ministry of Health considered using WISN but decided to develop their own method to estimate health workforce (Table 3). The main challenge in using WISN was the insufficient information for all its inputs. Therefore, the Ministry of Health opted to develop a context-specific tool to estimate the health workforce needed using its own available information resources such as the National Register of Health Personnel (INFORHUS computer application) [47].

New method developed: The method estimates the workforce gaps based on the resource need, availability and gap [51,52]. For the proper application of the methodology, MINSA provides technical assistance through the General Directorate of Health Personnel to all the stakeholders in the country including directorates of health, networks, hospitals and institutes, professional associations (nurses, midwives, technologists and others), the National Civil Service Authority, health (army, police) and to countries of the South American Gran Chaco and the Andean Region [51,52]. In order to make the tool user-friendly, the method uses matrix modules in Excel format and automated calculation of estimates.

This methodology has been used in Peru in accordance with the 2018–2030 health human resources policy guidelines [51,52]. It has been applied for various strategic decisions including (a) the public health investment projects, (b) strategy for the prevention and control of tuberculosis, (c) budget estimates for human resources in all the regional governments of the country, (d) planning of vacancies in the rural and urban marginal health service, and (e) training programs for medical specialists [38,39,40,41,42,44,45,51,52].

## 4. Discussion

This multi-country study presents three interconnected but unique experiences with WISN implementation. Lessons drawn from these countries will not only expand knowledge on WISN but will also assist with the effective roll-out and implementation of other global programmes or services. Even though WISN has been in existence since 1998, all the countries were not familiar with the earlier Excel-based WISN version. As a result, instead of building on prior knowledge, skills and infrastructure, this phase ended up being a catch-up for what would have possibly been achieved between 1998 and 2010. Secondly, both South Africa and India only implemented WISN in primary care due to lack of resources and infrastructure. Thirdly, none of the countries had information systems objectively measuring workload or working time. While South Africa received support from the WHO, this was limited in India as the country used the available modules without technical assistance.

While WISN assists with determining the ideal number of human resource needs, availability of funds for implementation of calculated human resource needs rendered the process futile in the context of South Africa. Over the years, a significant number of countries in the WHO African region and WHO SEARO regions have reported their experiences on using WISN with seemingly little uptake or reporting from Latin America [1,2,7,17,34,51,53,54,55,56,57,58,59,60,61]. However, the next steps after the implementation of WISN are not yet evident in these countries. Also, the time period for attaining this ideal number was not advised. In the light of epidemiological and demographic transition and with the recent COVID-19 pandemic, the ideal might never be achieved as it would be like chasing a moving target. While countries can support the education of health professionals, they do not have sufficient funds to retain all the healthcare workers within the public sector, as seen in South Africa [34,62,63]. Recent events in South Africa have demonstrated that there is limited consultation and planning on how excess human resources would be relocated.

### 4.1. Retention—An Important Factor for Human Resource Planning

WISN was developed in accordance with the basic goal of human resource management; that is, to have the right number of people, with the right skills, in the right place and at the right time [6]. While it has been a useful tool to plan human resources for health in several countries, it does not consider the motivation to perform and the extrinsic factors needed to increase retention. Historically, staffing requirements have been calculated as population-to-staff ratios. These methods do not consider the variations in the demand for services, task-sharing between team members, absenteeism and motivation to work in underserved areas. South Africa has various strategies to retain health professionals in rural areas which include (a) financial incentives, e.g., rural allowance, return-of-service schemes [32,63,64,65,66], (b) personal and professional support, e.g., provision of accommodation and opportunities to further their studies [32,66], (c) educational strategies, e.g., targeted recruitment for rural areas [32,66,67,68,69], and (d) regulatory strategies, e.g., compulsory community service for skilled health professionals [32,66,68,70]. The human resource retention strategies of the Peruvian Ministry of Health include economic compensations and deliveries in remote or border areas, emergency zones and for primary care, as well as non-financial compensations such as recognitions or distinctions for outstanding performance and personnel development [71].

### 4.2. Recommendations

WISN is an important tool for health workforce planning; however, it requires broader stakeholder consultation not just involving the health department, but also education, finance and other related ministries. This would thus align the implementation process with the WISN manual which recommends three implementation groups, namely, the steering committee, technical task force and expert working groups [6]. Secondly, it requires consultation with the health workforce. Ideally, the health workforce should be involved in the planning and implementation stage as this builds trust between the decision makers and the service providers. The WISN training modules should include details regarding the planning and consultation phase so that countries can use the tool without guidance of technical experts. Finally, health workforce planning should consider the country’s financial resources and the burden of disease.

### 4.3. Conclusions

In order to deliver quality, comprehensive primary health care to achieve the sustainable development goals and universal health coverage, detailed and robust health workforce planning is needed. WISN is a useful tool that has been used across several countries but must not be seen as a stand-alone solution. Planning and implementation require a comprehensive approach driven by an efficient health information system to implement its inputs. Innovative solutions, such as the method developed by the Peruvian Ministry of Health using available information is an example that can be adapted to other countries. Estimating the number of staff required is not enough; governments need to be able to afford their health workforce and, more importantly, be able to motivate, retain and sustain the health workforce for better health outcomes.

## Figures and Tables

**Table 1:** Country indicators [9,10,11,12].

Indicator	India	Peru	South Africa
Population, 2020	1.38 billion	32.63 million	59.62 million
GDP per capita (USD), 2019	2099.60	6977.69	6001.40
Human development index rank, 2019	131	79	114
Population density, 2020	420/km^2^	25/km^2^	49/km^2^
Income level, 2020	Lower-middle income	Upper-middle income	Upper-middle income
Physician density (per 1000 population), 2017	1.28	1.31	0.91
Nursing and midwifery personnel density, 2017	2.11	2.21	1.31
Life expectancy (years), 2018	69.4	76.5	63.9
Under-5 mortality rate, 2019	34.3	13.2	34.1

**Table 2:** Comparison of existing organogram posts to those recommended by WISN methods for a primary care setting of a South African province.

District	A: Number of Existing Posts *	B: Cost of Funded Posts (Rands)	C: WISN Determined Posts (B)	D: Cost of WISN Determination (Rands)
A	1513	574,016,100	2238	857,548,846
B	1888	775,264,389	2473	1,066,158,090
C	1693	641,221,482	2283	909,994,718
D	1803	684,681,481	2344	893,342,355
E	558	196,278,766	664	233,467,294
F	1504	579,318,958	2069	824,212,879
G	2231	901,029,019	2999	1,264,667,454
H	1135	411,370,294	1673	614,871,176
Total	12,325	4,763,180,488	16,743	6,664,262,812

Variance 1: C—A = 4418. Variance 2: D—B (Rands) = 1,901,082,324. Available budget (Rands) = 1,322,722,157. * These are funded posts but not necessarily filled.

**Table 3:** Comparison of WISN with Peruvian methodology to determine human resource for health [6,9,12,48,49,50].

Steps According to WISN	WISN	Methodology According to the Demand Approach	
	Conceptual Description	Conceptual Description	Input
Determine priority professional categories based on health facility type	Prioritises the occupational category including the health facilities.	Incorporates all health workers.	Portfolio of health services. Computer application National Registry of Health personnel.
Estimates available working time	Estimates available working time by occupational category discounting days of absenteeism.	Estimates available working time.	Annual contracted hours Vacation hours Uncompensated holiday hours Actual working hours
Define workload components	Defines workload groups and components (health service activities, support activities, additional activities).	Establishes two workload groups, assistance and non-assistance, assigning a percentage of available work time to each category.	Percentage of non-care activities (administrative activities, training, etc.). Percentage of care activities
Establish activity standards	Establishes the activity standard for each of the components or activities of the workload groups.	It establishes the standard time only of care activities for each occupational category involved in the service portfolio and other nursing staff scheduling criteria.	Standard of time per health service. Degrees of dependency and nursing care index (hospitalisation, critical care, among others). Percentage of distribution between nurses and nursing technicians.
Establish standard workloads	Estimates the amount of health services work that a worker can do in a year (if the total work time were dedicated exclusively to that activity).	It establishes the time per occupational category required to deliver all care activities in an annual period including the time required for critical services.	Annual effective demand of the health services portfolio. Time standard for health services.Time of attention in critical services
Calculate allocation factors	Estimates the staffing requirement to cover support activities using the category allocation factor and additional activities using the individual assignment factor.	Health personnel hours for administrative and training activities, among others, are estimated using an adjustment factor for the non-care component.	Percentage of non-care activities (administrative activities, training, etc.).
Determine staffing requirements based on the WISN	Determines the staffing requirement to meet all workload components of its category according to the previous year’s annual service statistics (annual workload). Uses rounding rules.	Estimates the need for health and non-health personnel to meet the projected annual effective demand. Uses rounding rules.	Effective annual demand of the health services portfolio. Rounding rules.
Analyse and interpret WISN results	Analyses the difference between the actual and the required number of personnel; as well as, examines the ratio between these two numbers (difference and ratio).	Similar analysis method. Adjustment of the need for health personnel according to population (for primary care). Uses Microsoft Excel format.	Gap and workload ratio of health care personnel

## Data Availability

Extra data are available by emailing the corresponding author.

## Strengths and Limitations of This Study

This study reports on the implementation experiences of the Workload Indicators for Staffing Needs (WISN) tool in three different countries and continents. This study also offers insights into an alternate health workforce planning strategy used in Peru. This study should help improve health workforce planning and WISN implementation experiences. A limitation of this study is that Peru opted to use a different health workforce planning tool when their assessment was that WISN would be contextually difficult to implement.
